# An R2R3-MYB Transcription Factor *RmMYB108* Responds to Chilling Stress of *Rosa multiflora* and Conferred Cold Tolerance of *Arabidopsis*

**DOI:** 10.3389/fpls.2021.696919

**Published:** 2021-07-27

**Authors:** Jie Dong, Lei Cao, Xiaoying Zhang, Wuhua Zhang, Tao Yang, Jinzhu Zhang, Daidi Che

**Affiliations:** ^1^College of Horticulture and Landscape Architecture, Northeast Agricultural University, Harbin, China; ^2^Horticultural Research Institute, Hangzhou Academy of Agricultural Sciences, Hangzhou, China

**Keywords:** *Rosa multiflora*, R2R3 MYB transcription factors, *MYB108*, cold tolerance, transgenic, *Arabidopsis*

## Abstract

A sudden cooling in the early spring or late autumn negatively impacts the plant growth and development. Although a number of studies have characterized the role of the transcription factors (TFs) of plant R2R3-myeloblastosis (R2R3-MYB) in response to biotic and abiotic stress, plant growth, and primary and specific metabolisms, much less is known about their role in *Rosa multiflora* under chilling stress. In the present study, *RmMYB108*, which encodes a nuclear-localized R2R3-MYB TF with a self-activation activity, was identified based on the earlier published RNA-seq data of *R. multiflora* plants exposed to short-term low-temperature stress and also on the results of prediction of the gene function referring *Arabidopsis*. The *RmMYB108* gene was induced by stress due to chilling, salt, and drought and was expressed in higher levels in the roots than in the leaves. The heterologous expression of *RmMYB108* in *Arabidopsis thaliana* significantly enhanced the tolerance of transgenic plants to freezing, water deficit, and high salinity, enabling higher survival and growth rates, earlier flowering and silique formation, and better seed quantity and quality compared with the wild-type (WT) plants. When exposed to a continuous low-temperature stress at 4°C, transgenic *Arabidopsis* lines–overexpressing *RmMYB108* showed higher activities of superoxide dismutase and peroxidase, lower relative conductivity, and lower malondialdehyde content than the WT. Moreover, the initial fluorescence (*F*_o_) and maximum photosynthetic efficiency of photosystem II (*F*_v_/*F*_m_) changed more dramatically in the WT than in transgenic plants. Furthermore, the expression levels of cold-related genes involved in the *ICE1 (Inducer of CBF expression 1)-CBFs (C-repeat binding factors)-CORs (Cold regulated genes)* cascade were higher in the overexpression lines than in the WT. These results suggest that *RmMYB108* was positively involved in the tolerance responses when *R. multiflora* was exposed to challenges against cold, freeze, salt, or drought and improved the cold tolerance of transgenic *Arabidopsis* by reducing plant damage and promoting plant growth.

## Introduction

Freezing temperatures induce cellular dehydration and limit the plant growth by inhibiting water uptake. Furthermore, chilling stress is fatal to plants, as cold thermodynamically lowers the membrane fluidity and directly inhibits several vital metabolic reactions (Mehrotra et al., 2020). In winter-hardy plants, the genes comprising a cryogenic response network mediate the physiological and biochemical changes and transcriptional modifications to maintain the cell integrity and plant survival at low temperatures (Zhao et al., 2015). We previously described that *Rosa multiflora*, a creeping thorny plant with medicinal and ornamental values, has a strong ability to withstand subzero temperatures (up to –40°C) in winter. The transcriptomic analysis of *R. multiflora* leaves exposed to different temperatures (25, 4, and –20°C) revealed that genes encoding APETALA2/ethylene-responsive factor (AP2/ERF), myeloblastosis (MYB), basic helix-loop-helix (bHLH), zinc finger protein (ZFP), NAC [NAM (NO APICAL MERISTEM), ATAF (Arabidopsis ACTIVATION FACTOR) and CUC (CUP-SHAPED COTYLEDON)] and WRKY transcription factors (TFs) actively participate in the response to cold treatment (Zhang et al., 2016). In plants, MYB TFs form one of the largest TF families, which are characterized by 1–4 incomplete conserved repeat (R)-containing DNA-binding domain located near the N-terminus (Klempnauer et al., 1982). Each R is composed of approximately 51–53 conserved amino acid residues with three α-helices (Dubos et al., 2010). The MYB family contains four types of TFs (i.e., 1R, R2R3, 3R, and 4R), depending on the number of MYB domains (Rogers and Campbell, 2004). The *R2R3-MYB* TFs form the largest clade and participate in the plant growth, especially in cell differentiation, development of floral organ, specific metabolisms, and response to environmental stress (Albert et al., 2014; Li et al., 2019; Liu et al., 2021).

In *Arabidopsis thaliana*, the *R2R3-MYB* TFs, such as AtMYB15, AtMYB30, AtMYB44, AtMYB96, and AtMYB108, are associated with stress responses (Dubos et al., 2010). The AtMYB15 protein interacts with the Inducer of C-repeat binding factor (CBF) Expression 1 (ICE1) and binds to Myb recognition sequences in the promoters of *CBF* genes to repress cold tolerance (Agarwal et al., 2006). In *Rosa chinensis*, *anthocyanidin synthase*, *flavonol synthase*, *orcinol O-methyltransferase 1* (*RcOOMT1*), and *RcOOMT2* genes are highly expressed in pink petals of flower buds and open flowers (Han et al., 2019). The silencing of *RcMYB84*/*RcMYB123* increases the susceptibility of *R. chinensis* to *Botrytis cinerea* and reduces the protective effects of treatment with jasmonic acid (JA; Ren et al., 2020). Moreover, the structural genes of proanthocyanidins and flavonoid have a high expression level in *RrMYB5-* and *RrMYB10*-overexpressed *Rosa rugosa* and tobacco (Shen et al., 2019). However, a complete understanding of how *R2R3-MYB* TFs respond to low-temperature stress in *Rosa* species is lacking.

Here, we identified the *R2R3-MYB* genes of *R. multiflora* that respond to low-temperature stress from its transcriptome data (Zhang et al., 2016). The functions of these genes were predicted based on the phylogenetic analysis of the homologs of *R. multiflora* and *Arabidopsis R2R3-MYB*. We further investigated the *RmMYB108* gene after combination of the RNA-seq data and prediction of the gene function. The overexpression (OE) of *RmMYB108* in *Arabidopsis* confirmed the role of *RmMYB108* in improving the tolerance against plant stress. This study provides a theoretical basis for cold-tolerance breeding in *Rosa* species.

## Materials and Methods

### Plant Materials and Growth Conditions

The cuttings of *R. multiflora* were obtained from the forest botanical garden of Heilongjiang in China (45.0°N, 128.4°E) and were grown under a 16-h light/8-h dark cycle at 25°C [control (CK)]. The 1-year-old cutting seedlings of *R. multiflora* were exposed to 4°C (CT1) in an artificial climate room or to −20°C (CT2) in a freezer to induce cold stress. Additionally, plants were either not watered or watered with 150 mM NaCl to imitate drought and salt stress, respectively. The leaves and roots of *R. multiflora* were collected and immediately frozen in liquid nitrogen.

*Arabidopsis thaliana* ecotype Columbia-0 was used for stable genetic transformation, and tobacco (*Nicotiana benthamiana*) was used for transient transformation. *Arabidopsis* plants were grown at 23°C under 12-h light/12-h dark cycle during the vegetative period and under 14-h light/10-h dark cycle during the reproductive phase.

### Bioinformatics Analysis of R2R3-MYB Proteins in *R. multiflora*

Putative R2R3-RmMYB proteins, which were queried with the MYB DNA-binding protein PF00249 in the Linux system HMMER 3.3.1 (<1E−10), were identified from a published RNA-seq data (SRA accession no.: PRJNA698412) and the whole genome sequence of *R. multiflora*^1^ (Jung et al., 2019). The coding sequences (CDSs) of *R2R3-MYB* genes in *A. thaliana* and amino acid sequences of the encoded proteins were downloaded from The Arabidopsis Information Resource (TAIR^2^). The sequence of *AtICE1* promoter was downloaded from the National Center for Biotechnology Information^3^, and the *cis*-acting elements of *AtICE1* promoter were predicted using PlantCARE^4^. The conserved MYB domains were defined by using SMART^5^ (Letunic and Bork, 2018). The sequence alignments were performed using DNAMAN. A phylogenetic analysis was carried out using the maximum likelihood method with 1,000 bootstrap replicates in MEGA X. The heatmap was generated by using the Toolbox for Biologists (TBtools) software; the value in the row scale was normalized by the equation: $value=\frac{X-\mu }{\sigma }$, *X* is fragments per kilobase per million (FPKM), *μ* is mean, and *σ* is standard deviation of all FPKM in the row (Chen C. et al., 2020).

### Quantitative Real-Time Polymerase Chain Reaction

The levels of gene expression were determined by using the quantitative real-time polymerase chain reaction (qRT-PCR), as described earlier (Zhang et al., 2016). The comparative Ct method was used to analyze the level of gene expression (Schmittgen and Livak, 2008). Primers used for qRT-PCR are listed in Supplementary Table 1. *RmUBC* (Klie and Debener, 2011) and *AtActin2* (Zhou et al., 2018) were selected as reference genes in *R. multiflora* and *Arabidopsis*, respectively. All experiments were performed in three biological replicates, each containing three technical repeats.

### Subcellular Localization of RmMYB108

The *RmMYB108* CDS without the stop codon was amplified by PCR and cloned into the pBI121-green fluorescent protein (GFP) vector using ClonExpress II One Step Cloning Kit (Vazyme Biotech Co., Ltd., Nanjing, China) to generate *35S*::*RmMYB108*-*GFP* construct. The primers used for plasmid construction are listed in Supplementary Table 1. The *35S::RmMYB108-GFP* plasmid (Addgene: 173181) and *35S::GFP* [positive control (PC)] plasmid were introduced into *Agrobacterium tumefaciens* strain GV3101, which was then injected into the leaves of 1-month-old *N. benthamiana* plants. The subcellular localization of *35S*::*RmMYB108*-*GFP* and 35S::GFP was visualized by confocal laser scanning microscopy (TCS SP8, Wetzlar, Germany) 3 days post infiltration.

### Transcriptional Activation of *RmMYB108*

The full-length CDS of *RmMYB108* was introduced into the pGBKT7 vector using *Nde*I and *Bam*HI restriction endonucleases and T4 ligase (Thermo Fisher Scientific, Waltham, MA, United States). The primers that were used for plasmid construction are listed in Supplementary Table 1. *pGBT9* was used as a PC that could activate *HIS3*, *ADE2*, and *MEL1* reporter genes and X-α-Gal activity in the transformed yeast cells on the medium lacking tryptophan (Trp) and histidine (His). *pGBKT7* was used as a negative control (NC). The NC, PC, and *pGBKT7-RmMYB108* plasmids (Addgene: 173183) were transformed into the yeast strain AH109 and cultivated on synthetic-defined (SD)/-Trp or SD/-Trp/-His medium with or without X-α-Gal.

### Vector Construction and Plant Transformation

To construct *35S::RmMYB108*, the *RmMYB108* CDS was amplified using the primers listed in Supplementary Table 1, and then the PCR product was ligated into the *Kpn*I and *Bam*HI sites of the pCAMBIA1301 vector downstream to the *35S* promoter of the cauliflower mosaic virus (Addgene: 173180). The resulting vector was introduced into *A. tumefaciens* strain GV3101.

To generate *RmMYB108*-overexpressing lines, *Arabidopsis* plants were transformed with the *35S::RmMYB108* construct using the floral dipping method (Clough and Bent, 1998). A semiquantitative reverse-transcription PCR (RT-PCR) assay was performed to select T_2_ generation, which was obtained from healthy T_1_ plants grown on the half-strength Murashige and Skoog (1/2 MS)–agar medium containing 30 mg/L Hygromycin. Homozygous *RmMYB108* OE lines (#7, #9, and #11) in the T_3_ generation were further identified by screening of Hygromycin resistance and PCR.

### Stress Treatments of *Arabidopsis*

For induce stress as oxidation, dehydration, and salt, sterilized seeds or 1-week-old seedlings of *Arabidopsis* were grown in 1/2 MS medium containing 3% sugar and 0.75% agar, which was further supplemented with or without 1.2 mM hydrogen peroxide (H_2_O_2_), 150 mM mannitol, or 150 mM NaCl. The plates were incubated under 12-h light/12-h dark cycle at 23°C vertically. To induce cold stress, the sterilized seeds and 1-week-old seedlings in plates without any supplement were incubated in a growth chamber that was maintained at 15 or 4°C vertically. The seed germination and seedling growth were observed and measured after cultivating for 1 and 2 weeks, respectively. To test the freezing tolerance of OE lines, the 20-day-old *Arabidopsis* plants were cold-acclimated at 4°C for 12 h and then subjected to −10°C for 2.5 or 4 h. Subsequently, the plants were subjected again to 4°C for 12 h and then grown at 23°C.

### Physiological Measurements of Plants

The leaves of 20-day old wild-type (WT) plants and *RmMYB108* OE lines were collected after exposure to 4°C for 0, 1, 3, 6 12, and 24 h. The relative conductivity (RC), superoxide dismutase (SOD) activity, peroxidase (POD) activity, and malondialdehyde (MDA) content of plant leaves were measured as described earlier (Miao et al., 2010).

### Measurement of Chlorophyll Fluorescence

The 20-days-old WT and transgenic *Arabidopsis* plants were exposed to 4°C for 0, 1, 3, 6, 12, and 24 h; the rosette leaves of *Arabidopsis* were used for measuring chlorophyll fluorescence. The initial fluorescence (*F*_o_) and the highest electronic efficiency (*F*_v_/*F*_m_) of photosystem II (PS II) were measured using IMAGING-PAM (Walz, Germany), according to the instructions of the manufacturer. The same leaf position was used for measurements in both plant groups.

### Statistics and Analysis

All data were subjected to ANOVA, followed by the least significant difference test. All statistical analyses were performed using IBM SPSS (New York, NY, United States) software. The results were displayed by graphs and charts using GraphPad Prism 8.0.

## Results and Analysis

### R2R3-RmMYBs Potentially Regulate Plant Growth, Development, and Stress Tolerance in *R. multiflora*

The analysis of *R. multiflora* RNA-seq data (SRA accession no.: PRJNA698412) revealed many differentially expressed genes, which encoded TFs involved in metabolism, transcription, transport, and signal transduction in response to cold stress. For example, some genes belonging to the *AP2/ERF*, *MYB*, and *NAC* families were expressed in higher levels at both 4°C (CT1) and −20°C (CT2) than at 25°C (CK); however, the specific functions of most of these genes are unknown. In this study, we focused on how the R2R3-RmMYBs respond to low-temperature stress in *R. multiflora*. Based on the FPKM data, 37 *R2R3-RmMYB* genes (Supplementary Table 2 and Supplementary Figure 1) exhibited three expression trends in response to cold treatment. Genes including *RmMYB018* and *RmMYB44b* were upregulated in the CT1 and CT2 treatments, while genes such as *RmMYB308c* and *RmMYB24a* were downregulated in both treatments. Several genes including *RmMYB35* and *RMMYB106* were induced only at CT1.

**FIGURE 1:**
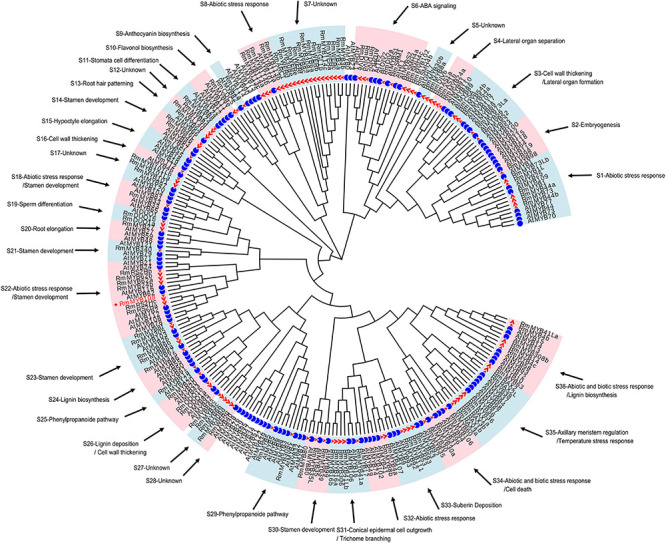
Putative functions of *R2R3*-*MYB* genes in *Rosa multiflora* based on the cluster results between *R. multiflora* and *Arabidopsis thaliana* in the phylogenetic tree. Red triangle marked *R2R3-MYBs* in *R. multiflora* and blue circle represented *R2R3-MYBs* in *A. thaliana*. The circular unrooted tree was generated by maximum likelihood method. Different subgroups were marked with pink and light blue background, and the function of each subgroup referred the genes with confirmed functions in *A. thaliana*.

To understand the possible function as well as identifying potential chilling stress-responsive genes in this family, 119 R2R3-MYBs with complete sequences, which were extracted from 2R subgroup (Supplementary Table 3), were identified and named by referring to the protein BLAST results and the naming principle of *R2R3-MYB* in *R. chinensis* (Han et al., 2019; Supplementary Figure 2). Notably, the TFs with identical names were differentiated by lowercase letters; these MYB TFs showed extremely similar sequences and blastp results (Supplementary Table 4). According to the function of 134 *Arabidopsis R2R3-MYBs* (Supplementary Table 5), 114 *R2R3-MYBs* in *R. multiflora* were divided and clustered to 30 function-annotated and six function-unknown subgroups, with the exception of 5 *R2R3-MYBs*, which shared low sequence similarity with AtMYBs in the ML phylogenetic tree (Figure 1). Based on their functional annotation, *RmMYBs* were divided into three classes. *RmMYBs* in class I were further divided into eight subgroups (i.e., S9, S10, S16, S24, S25, S26, S29, and S33) and were involved in specific metabolisms, such as the regulation of lignin, anthocyanin, and flavonol biosynthesis. For example, *RmMYB8e* clustered closely with *AtMYB11* and *AtMYB12*, which modulate flavonoid biosynthesis in favor of flavonol accumulation (Pandey et al., 2015; Wang et al., 2016). Class II contained 14 subgroups (i.e., S2, S3, S4, S11, S13, S14, S15, S19, S20, S21, S23, S30, S31, and S35), and *RmMYBs* in these subgroups played important roles in the fate of the plant cell, especially in the regulation of axillary meristem and stamen development. Class III *RmMYBs*, which were divided into eight clades (i.e., S1, S6, S8, S18, S22, S32, S34, and S36), were involved in the response to biotic and abiotic stress. In class III, some *RmMYBs* showed a close relationship with *Arabidopsis R2R3-MYBs* involved in stress tolerance, such as *AtMYB21* (Zhang et al., 2021), *AtMYB44* (Jung et al., 2007), or *AtMYB108* (Cui et al., 2013). Furthermore, some *R2R3-MYB* genes involved in the response to abiotic and biotic stress were also involved in the regulation of stamen development or specific metabolisms. These results suggest that *R. multiflora R2R3-MYBs* perform a wide range of functions including the regulation of growth and development of rose plants, as well as its tolerance to harsh environments.

### *Rosa multiflora R2R3-MYB* Genes Response to Chilling Stress

**FIGURE 2:**
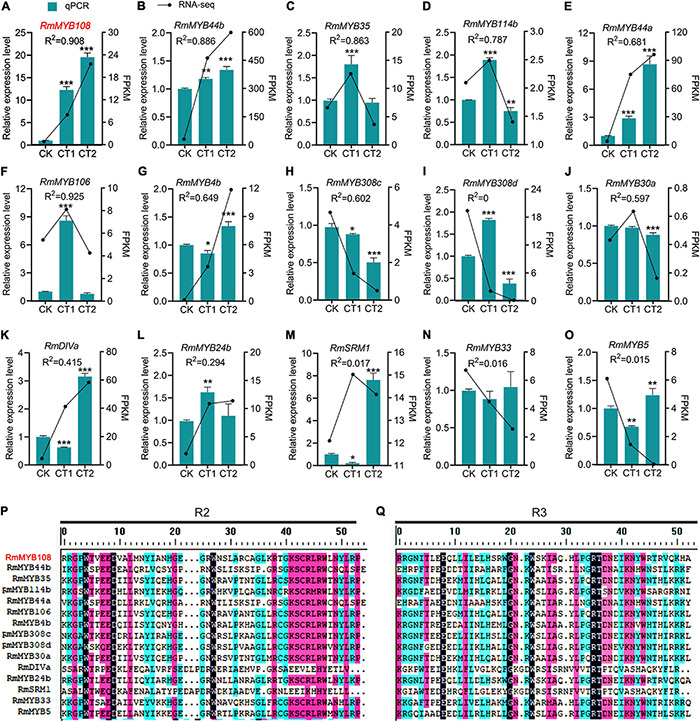
*R2R3-MYBs* in *R. multiflora* respond to chilling stress. Relative expression level and fragments per kilobase per million (FPKM) of **(A)** *RmMYB108*, **(B)** *RmMYB44b*, **(C)** *RmMYB35*, **(D)** *RmMYB114b*, **(E)** *RmMYB44a*, **(F)** *RmMYB106*, **(G)** *RmMYB4b*, **(H)** *RmMYB308c*, **(I)** *RmMYB308d*, **(J)** *RmMYB30a*, **(K)** *RmDIVa*, **(L)** *RmMYB24b*, **(M)** *RmSRM1*, **(N)** *RmMYB33*, **(O)** *RmMYB5* under 25°C (CK), 4°C (CT1) and −20°C (CT2). The correlation coefficient of each gene between FPKM from RNA-seq and relative expression level from quantitative real-time polymerase chain reaction (qRT-PCR) was represented by *R*^2^ (coefficient of determination). *p < 0.05; **p < 0.01; and ***p < 0.001 in statistics. The aligned sequences of both R2 **(P)** and R3 **(Q)** domains of 15 R2R3-MYB from *R. multiflora* were evolutionarily highly conserved.

The *R. multiflora* RNA-seq data analysis and prediction of gene function revealed a total of 15 stress-responsive *R2R3-MYB* genes (Figure 2). The gene expression analysis by using the qRT-PCR confirmed that *RmMYB108, RmMYB44b*, and *RmMYB44a* were upregulated in response to chilling stress, while *RmMYB106*, *RmMYB35*, *RmMYB114b*, and *RmMYB308c* were upregulated at 4°C and downregulated at –20°C. Moreover, the expression trends of the abovementioned genes showed a strong correlation with the RNA-seq results. However, while some other *R. multiflora* genes such as *DIVARICATA* (*RmDIVa*), *RmMYB308d*, and *Salt-Related MYB1* were differentially expressed under low-temperature treatment, their correlation between qRT-PCR and RNA-seq data was not significant (Figures 2A–O). The alignments of the amino acid sequence of the R domains of these 15 *R2R3-MYB*s showed that the Myb domains of R2 and R3 were evolutionarily highly conserved, with spacer sequences [-W-(X19)-W-(X19)-W-…-F/I-(X18)-W-(X18)-W-] (Figures 2P,Q).

Thus, the results of the prediction of gene function together with RNA-seq data revealed that the 15 *R. multiflora R2R3-MYB* genes are responsive to the cold treatment. Among those genes, *RmMYB108* showed the highest relative expression level, and its expression trend was fitted with the transcriptome result under low-temperature treatment. In addition, the effect of *RmMYB108* on plant cold tolerance has not been investigated to date although the phylogenetic tree showed that *RmMYB108* has a close relationship with *R2R3-MYBs* of *Arabidopsis* which are involved in stress resistance. Therefore, we further explored the potential role of *RmMYB108* in cold tolerance in *Arabidopsis*.

### Molecular Characterization of *RmMYB108*

**FIGURE 3:**
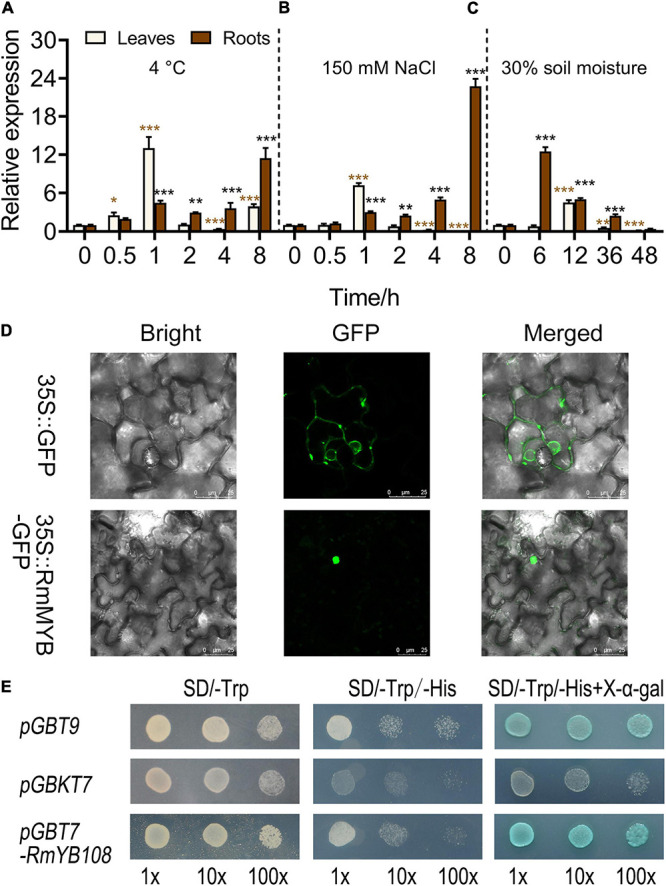
Analysis of gene expression, localization, and transactivation assay of the *RmMYB108* transcription factor. *RmMYB108* expression patterns in *R. multiflora* leaves and roots responding abiotic stress under continuous 4°C **(A)**, 150 mM NaCl **(B)**, and drought **(C)** treatment. **(D)** The positive control *35S::GFP* and *35S::RmMYB108* constructs were expressed in tobacco epidermal cells, showing the nuclear localization of *RmMYB108*. **(E)** Transcriptional activation assay of *RmMYB108*. Growth of yeast cells (strain AH109) transformed with pGBT9 vector (a positive control), pGBKT7 vector (a negative control), or pGBKT7-*RmMYB108* vector on SD/-Trp or SD/-Trp/-His with or without X-α-gal. 10×, 10×, and 100× represent yeasts were diluted with original concentration (OD600 = 0.4), 10–, 100-folds, respectively. *p < 0.05; **p < 0.01; ***p < 0.001 in statistics.

The expression of *RmMYB108* was first investigated under stress due to cold, salt, and drought using the qRT-PCR. At 4°C, *RmMYB108* expression in rose leaves was induced within 0.5–2 h of the treatment, peaked at 1 h, and then decreased to its original level at 2 h. Furthermore, under cold stress, *RmMYB108* showed a greater increase in the expression in roots over time, with the highest value at 8 h (Figure 3A). Notably, the expression pattern of *RmMYB108* under salt stress (150 mM NaCl) was similar to that under cold stress (Figure 3B). In the drought treatment, the *RmMYB108* expression peaked at 6 h in roots and at 12 h in leaves (Figure 3C). Thus, the expression of *RmMYB108* in rose leaves and roots was affected by stress due to cold, salt, and drought at different time points. Interestingly, these results suggest that rose leaves sense environmental signals earlier than roots, although roots potentially exhibited a greater tolerance to stress than leaves.

To determine the subcellular localization of the *RmMYB108* protein, the *RmMYB108* CDS was fused with the N-terminus of the *GFP* gene. As expected, *RmMYB108* was localized to the nucleus in tobacco epidermal cells (Figure 3D). In addition, the experiments conducted in yeast confirmed that the full-length *RmMYB108* exhibited self-activation, as the yeast AH109 cells transformed with the *pGBKT7-RmMYB108* and *pGBT9* (PC) grew well on the SD/-Trp/-His medium and turned blue on the selective medium containing X-α-Gal (Figure 3E).

### Overexpression of *RmMYB108* Enhanced the Cold Tolerance of *Arabidopsis* at the Early Growth Stage

**FIGURE 4:**
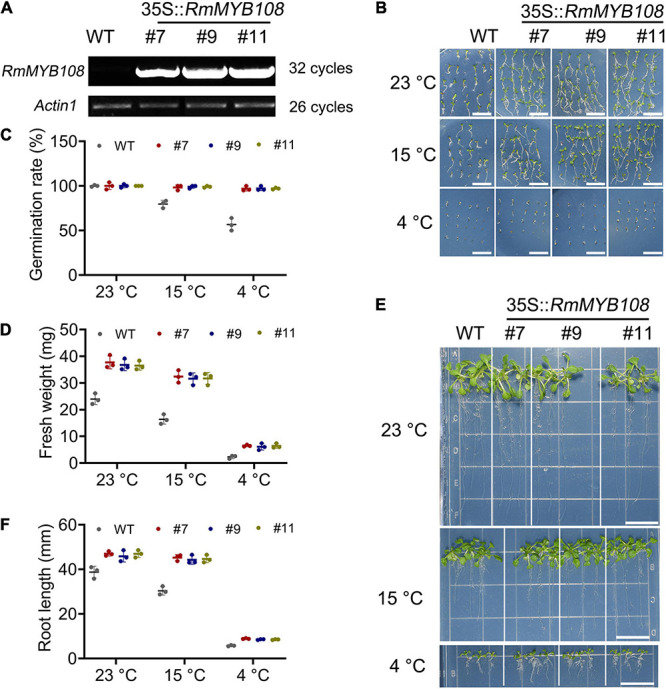
Identification of *RmMYB108*-overexpressing *Arabidopsis* and cold tolerance contrast between wild type (WT) and #7, #9, and #11 OE lines at early growth stage of plants. **(A)** semiquantitative RT-PCR analysis of *RmMYB108* and *Actin1* in WT and overexpression (OE) lines showed the overexpression of *RmMYB108* in transgenic plants. The 7-days seed germination phenotype **(B)**, seed germination rate **(C)**, 21-day seedlings fresh weight **(D)**, growth **(E)**, and root length **(F)** of WT and OE lines under 23, 15, and 4°C, respectively, indicated OE lines had higher cold tolerance than WT. Data represent mean value + *SD* (*n* = 30). *p < 0.05; **p < 0.01; ***p < 0.001 in statistics.

To characterize the function of *RmMYB108*, pCAMBIA1301-*RmMYB108* was transformed into *Arabidopsis via* the *Agrobacterium*-mediated transformation. Three *RmMYB108* OE lines (#7, #9, and #11) were identified by using the semiquantitative RT-PCR (Figure 4A). We then compared the growth and cold tolerance of WT and *RmMYB108* OE lines at the stages of germination and seedling. The rate of seed germination of the WT was similar to that of the OE lines under normal (no stress) conditions but reduced significantly at 15 and 4°C. While 98% of the transgenic seeds stayed alive, more than 40% WT seeds showed no germination at 4°C (Figures 4B,C). Interestingly, the growth vigor of seedlings (determined by their fresh weight and primary root length) was weaker in the WT than in OE lines even at 23°C, and the growth potential of WT seedlings was affected more dramatically than that of OE lines with the decrease in temperature (Figures 4D–F). At 14 days after treatment, the weight of WT seedlings decreased by 31.78% at 15°C and by 90.27% at 4°C, whereas that of OE lines decreased by 13.6 and 82.78%, respectively. These results suggest that the OE of *RmMYB108* in *Arabidopsis* accelerated the plant growth as well as enhanced tolerance to chilling temperatures by alleviating stress-induced inhibition of seed germination and plant growth.

### Overexpression of *RmMYB108* Enhanced Cold Tolerance at Maturity

**FIGURE 5:**
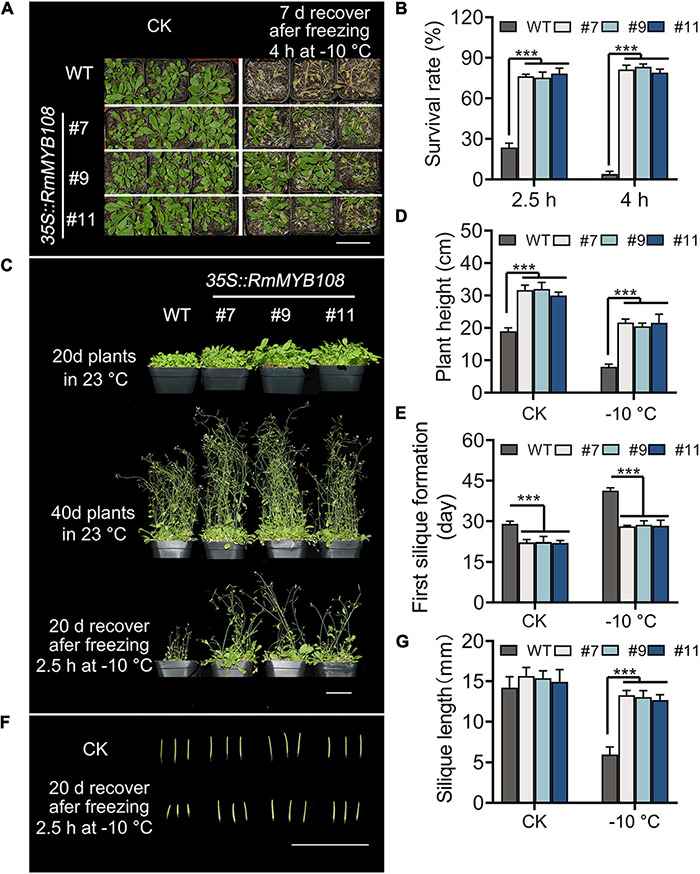
Phenotype of transgenic *RmMYB108* plants after shortly freezing at –10°C at vegetative and reproductive growth stages of plants. **(A)** Phenotype of 20-days-old WT and OE lines that were treated at –10°C for 4 h and then recovered for 7 days. **(B)** Survival rate of recovered plants after being frozen at –10°C. The 20-day WT and OE lines were pictured after freezing 2.5 h at –10°C and recovering after 20 days **(C)**, the plant height **(D)**, first silique formation time **(E)**, and silique length **(F,G)** were measured to show that the overexpression of *RmMYB108* enhanced cold tolerance at maturity. Data represent mean value + *SD* (*n* = 20). *p < 0.05; **p < 0.01; ***p < 0.001 in statistics.

The cold tolerance assays were further carried out in mature plants of the WT and OE lines. When exposed to –10°C for 4 h, transgenic plants showed a higher survival rate and greater tolerance against freezing stress than WT plants, with several yellow leaves (Figure 5A), whereas WT plants showed the most withered leaves and less than 15% survival (Figure 5B). When the duration of freezing stress was shortened to 2.5 h, the survival rate of WT plants increased to 20%, while that of OE lines was maintained at approximately 80% (Figures 5B,C). Forty-day-old transgenic plants were more robust than WT plants with or without cold treatment. A short exposure to freezing temperatures greatly harmed WT plants, as evident from the sharp reduction in the plant height from 19 to 8 cm, whereas OE lines suffered less damage, as the plant height decreased only from 31.22 to 21.27 cm (Figure 5D). In addition, flowering and silique formation occurred earlier in OE lines than in the WT under CK and cold conditions. The first silique appeared at 29 days in the WT and 22.5 days in OE lines under normal conditions but at 41.33 and 28.43 days, respectively, under cold conditions (Figure 5E). Furthermore, after freezing, silique length of the WT was only 5.95 cm, which is much shorter than that of OE lines with 13.00 cm at average (Figures 5F,G). These results suggest that the OE of *RmMYB108* in *Arabidopsis* improved cold tolerance of the plant and shortened the plant growth cycle even after freezing.

### Overexpression of *RmMYB108* Induced Physiological Changes in *Arabidopsis*

**FIGURE 6:**
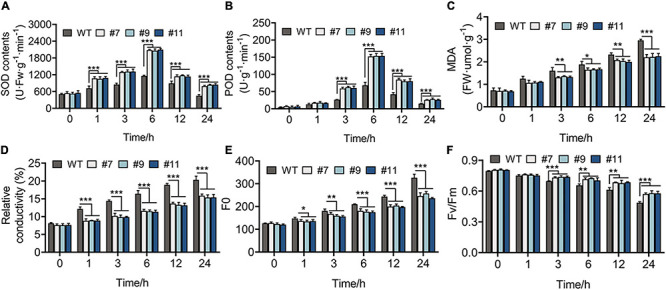
Physiological indicators of 20-days-WT and OE lines that were treated under 4°C for 0, 1, 3, 6, 12, and 24 h, respectively. **(A)** Superoxide dismutase (SOD) activity, **(B)** peroxidase (POD) activity, **(C)** malondialdehyde (MDA) content, **(D)** relative conductivity, **(E)** initial fluorescence (*F*_o_), **(F)** the biggest electronic efficiency (*F*_v_/*F*_m_) changes between WT and OE lines provided an evidence for the growth of OE lines was less restricted than WT under chilling stress. *p < 0.05; **p < 0.01; ***p < 0.001 in statistics.

The healthy WT and OE lines were grown under the treatment at 4°C for 1 week, while the leaves of these plants were green, with no indication of wilting or dehydration. The physiological changes were observed in plants within 24 h of treatment at 4°C and indicated that all the physiological indexes of WT plants and OE lines showed the same variational trend under cold treatment in the vegetative phase. The SOD and POD activities of *Arabidopsis* peaked at 6 h after chilling stress, and their values were higher in OE lines than in WT plants at each time point, except at 0 h. For example, compared with the control at 6 h, SOD activity increased by 128.08% in the WT and by 284.20, 291.96, and 281.87% in OE lines #7, #9, and #11, respectively (Figures 6A,B). In addition, MDA content and RC, which exhibit a negative correlation with cold tolerance, showed a greater increase in WT plants than in OE lines. At 24 h, the RC of WT lines increased by 20%, whereas that of OE lines increased by an average of 15.54% (Figures 6C,D). Furthermore, the value of *F*_*o*_, which shows a linear relationship with the content of photosynthetic pigment, increased more in WT plants than in OE lines under chilling stress (Figure 6E). The value of *F*_v_/*F*_m_ decreased over time from 0.795 to 0.490 in the WT, 0.806 to 0.570 in line #7, 0.809 to 0.579 in line #9, and 0.805 to 0.577 in line #11 (Figure 6F).

### *RmMYB108* Improves Plant Cold Tolerance by Upregulating the CBF Cascade

**FIGURE 7:**
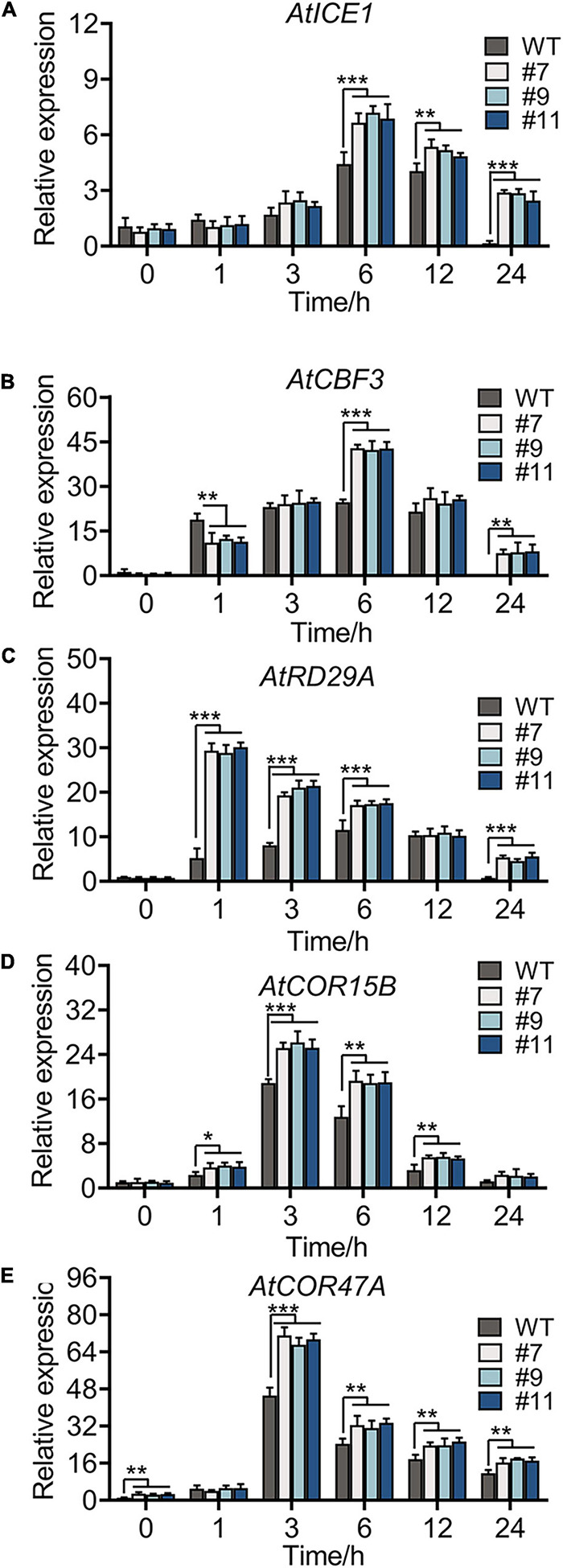
Cold-responsive marker genes expression level of WT and OE lines under 4°C for 0, 1, 3, 6, 12, and 24 h, respectively. Relative expression level of *AtICE1* **(A)**, *AtCBF3* **(B)**, *AtRD29A* **(C)**, *AtCOR15B* **(D)**, and *AtCOR47A* **(E)** were measured to reveal that *RmMYB108* probably improved plant tolerance by CBF-dependent under cold treatment. *p < 0.05; **p < 0.01; ***p < 0.001 in statistics.

The expression of some marker genes involved in cold tolerance was tested in the WT and OE lines under chilling stress. Genes, such as *AtICE1*, *AtCOR47A*, *AtCBF3*, *AtCOR15B*, and *AtRD29A* (Zhou et al., 2011; Thalhammer and Hincha, 2014), in the *ICE1-CBF-COR* cascade showed a similar expression in the WT and OE lines under normal conditions. Under chilling stress, all genes were upregulated, reaching peak values at 3 or 6 h (Figures 7A–E), with more drastic changes in OE lines than in the WT. No phenotypic difference was apparent between WT plants and OE lines at continuous 4°C; however, the accumulation of oxides and loss of permeability of the cell membrane were observed in plants of all genotypes. Notably, compared with WT plants, all OE lines showed a minimal injury with a similar trend in physiological indexes and expression of genes related to cold resistance.

### Overexpression of *RmMYB108* Decreases Sensitivity to Other Stress

**FIGURE 8:**
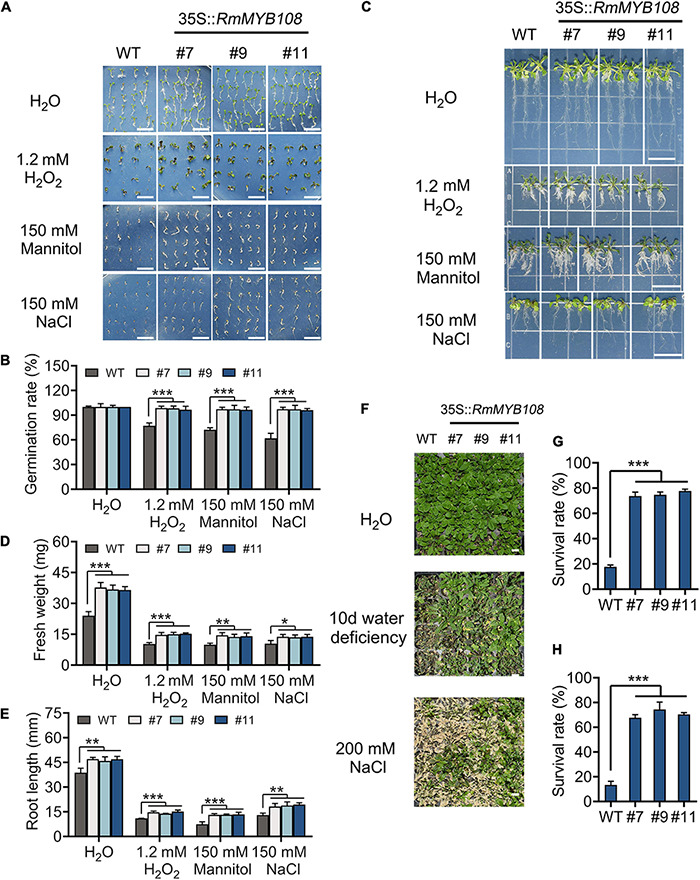
Effects of stress due to simulated oxidation, drought, and salt on plant growth. Seed germination phenotype **(A)** and germination rate **(B)**, seedlings growth **(C)**, fresh weight **(D)**, and root length **(E)** of WT and OE lines were observed and measured under deionized water (H_2_O), 1.2 mM H_2_O_2_, 150 mM mannitol, and 150 mM NaCl, respectively. The 20-days WT and #7, #9, and #11 were not watered or watered with 200 mM NaCl for 10 days **(F)**; the survival rate **(G,H)** were calculated and revealed that *RmMYB108* enhanced plant tolerance against stress due to dehydration and oxidation. Data represent mean value + SD (*n* = 30). *p < 0.05; **p < 0.01; ***p < 0.001 in statistics.

The seed germination and seedling growth of *Arabidopsis* were investigated under other stress to exemplify the essential role of *RmMYB108* in abiotic stress tolerance. In deionized water, WT and transgenic seeds showed the same germination rate (Figure 8A). However, in the presence of 1.2 mM H_2_O_2_, 150 mM mannitol, or 150 mM NaCl, the germination rate of WT seeds decreased to 50–70%, while that of OE lines #7, #9, and #11 remained over 95% (Figure 8B). Furthermore, the vigor of WT seedlings was lower than that of OE lines in all treatments. The fresh weight and primary root length of WT seedlings were significantly lower than those of OE lines in the treatment of stress due to oxidation, drought, and salt (Figure 8C). Under normal conditions, the average primary root length of WT seedlings was 38.79 mm, while that of OE lines was approximately 46.63 mm; however, when treated with 1.2 mM H_2_O_2_, 150 mM mannitol, and 150 mM NaCl, the average primary root length decreased to 11, 7.47, and 13 mm in the WT and averaged to 14.59, 13.27, and 18.82 mm in the OE lines, respectively (Figures 8D,E). Additionally, water deficiency or 200 mM NaCl treatments for 10 days significantly damaged the growth and development of *Arabidopsis* plants (Figure 8F). The survival rate of WT was less than 20% in both salt and drought treatments after returning to normal growth conditions, while that of OE lines was maintained at 60–80% (Figures 8G,H). These phenomena revealed that *RmMYB108* enhanced plant tolerance to stress due to dehydration and oxidation.

## Discussion

The *R2R3-MYB*s play essential roles in responses of the plant to abiotic stress. *MYB* genes in wheat [*Triticum aestivum*; *TaMYB31* and *TaMpc1-D4* (*Myb protein colorless 1 located on chromosome D*)] and cotton (*Gossypium hirsutum*; *GaMYB85*) respond to drought stress (Zhao et al., 2018; Li et al., 2020). Similarly, the *Salt Stress Regulator 1* (*PtrSSR1*) gene in poplar (*Populus trichocarpa*), *AcoMYB4* in pineapple (*Ananas comosus*), and *SiMYB305* in sesame (*Sesamum indicum*) mediate the tolerance against stress due to salt or drought through abscisic acid (ABA) signaling (Fang et al., 2017; Chen H. et al., 2020; Dossa et al., 2020). In apple (*Malus domestica*), *MdMYB308L*, *MdMYB23*, *MdMYB88*, and *MdMYB124* regulated cold hardiness *via* CBF-dependent and CBF-independent pathways (An et al., 2018; Xie et al., 2018; An et al., 2020). In this study, we found that *RmMYB108*, *RmMYB44a*, and *RmMYB44b* participated in the cold tolerance response of *R. multiflora* (Figure 1). The *ICE1-CBF-COR* gene cascade has been shown to contribute to cold acclimation by protecting plants from freezing damage in *Arabidopsis* (Zhao et al., 2015). *AtICE1*, *AtCBF3*, *AtRD29A*, *AtCOR15B*, and *AtCOR47A* were upregulated in *Arabidopsis* within 24 h under chilling stress, and *RmMYB108* OE lines showed the higher expression levels of these genes than those in WT plants (Figure 7). Furthermore, the promoter of *ICE1* harbors MYB-binding sequences (Supplementary Table 6). Therefore, we predicted that *RmMYB108* enhances cold tolerance probably *via* the CBF-dependent pathway in *Arabidopsis* (Supplementary Figure 3).

*RmMYB108* was upregulated in the transcriptome of *R. multiflora* in response to low-temperature stress, improved the ability to resist abiotic stress, and accelerated the growth of *Arabidopsis* plants by regulating changes in proteins in the cytoplasm or by indirectly maintaining the integrity of the cell membrane under stress. The stress due to low temperature, drought, and salt limits the availability of water to plant cells (Khan et al., 2018). This stress can cause membrane rupture and the outward flow of ions due to difference in osmotic pressure between the intra- and extracellular spaces, leading to plasmolysis and even cell death (Djemal and Khoudi, 2016; Ritonga and Chen, 2020). *RmMYB108*, which acts as a TF in the nucleus, regulates the expression of downstream genes probably by binding to their promoter regions on receiving the signals due to abiotic stress transmitted by the receptors. These genes generally participate in the minimization of oxidative damage, ion and water balance, osmotic stress response, and specific metabolisms to protect plant cells from water-related stress (Figure 6; Khan et al., 2018). Once the culture conditions were appropriate, the OE lines recovered and blossomed faster than WT obviously.

The recent studies showed that *MYB108* orthologs are associated with plant development, specific metabolisms, and stress response. MYB108 is believed to be a JA-responsive TF involved in the development of stamen and pollen and defense signaling in plants (Mandaokar and Browse, 2009; Cheng et al., 2016; Xu et al., 2019). *RmMYB108* has a close relationship with *AtMYB112*, *AtMYB78*, and *AtMYB108* in *Arabidopsis* (Figure 1 and Supplementary Figure 2); *AtMYB112* responds to salinity and high-light stress (Lotkowska et al., 2015), *AtMYB78* belongs to ABA-related genes (Sun et al., 2020), and *AtMYB108* acts as a negative regulator of ABA-induced cell death, (Cui et al., 2013). In *Prunus mum*e, *PmMYB108* (90% sequence similarity with *RmMYB108*) is positively associated with organ color (Zhang et al., 2018). In *R. chinensis*, *RcMYB108*, which is homologous to *RmMYB108* (94.17% sequence similarity), was upregulated during petal senescence and shedding, and silencing of *RcMYB108* altered the expression of senescence-associated genes and blocked ethylene- and JA-induced petal senescence (Zhang et al., 2019). Interestingly, when *Arabidopsis* seeds were cultured at 4°C for 6 months, we found that the speed of seed germination and plant growth was greatly reduced (data not shown). The germination rate of WT seeds was very low, whereas the seeds of most of the OE lines germinated well, and the resulting seedlings subsequently bloomed under harsh environments. After transferring to normal conditions, the transgenic plants grew well and quickly formed siliques. By contrast, WT seedlings showed a very low survival rate. Based on the evaluation of stress tolerance of OE lines and WT plants, we speculated that the *RmMYB108* gene promotes cell division and differentiation to facilitate plant senescence, shortens the plant growth cycle, and enhances tolerance against abiotic stress.

In addition to an increasing plant yield, plant breeders focus on how to shorten the breeding period and increase the environmental stress resistance of plants (Bhatta et al., 2021). These breeding goals can generally be achieved through the genetic modification of plants by targeting genes encoding TFs (Hoang et al., 2017; Khan et al., 2018; Nowicka et al., 2018; Baillo et al., 2019). In this study, OE of *RmMYB108* in *Arabidopsis* promoted the tolerance against stress due to drought, salt, and freezing of transgenic plants, manipulated their growth cycle, and increased their biomass and seed yield under cold stress. This approach can be used to accelerate wood growth, reduce the length of the plant breeding cycle, enhance stress tolerance, and improve land utilization by developing more productive and resilient crops that can feed the global population, which is predicted to reach 10 billion by 2050 (Bhatta et al., 2021).

## Data Availability Statement

The datasets presented in this study can be found in online repositories. The names of the repository/repositories and accession number(s) can be found in the article/Supplementary Material.

## Author Contributions

JD designed and performed the experiments, interpreted the data, and wrote the article. DC, JZ, and LC designed the experiments and edited the article. XZ performed the experiments. WZ and TY analyzed the data. All authors contributed to the article and approved the submitted version.

## Conflict of Interest

The authors declare that the research was conducted in the absence of any commercial or financial relationships that could be construed as a potential conflict of interest.

## Publisher’s Note

All claims expressed in this article are solely those of the authors and do not necessarily represent those of their affiliated organizations, or those of the publisher, the editors and the reviewers. Any product that may be evaluated in this article, or claim that may be made by its manufacturer, is not guaranteed or endorsed by the publisher.

## Footnotes

1
https://www.rosaceae.org/

2
http://www.arabidopsis.org/

3
https://www.ncbi.nlm.nih.gov/

4
http://bioinformatics.psb.ugent.be/webtools/plantcare/html/

5
http://smart.embl-heidelberg.de/

## Supplementary Material

Supplementary Figure 1 The heatmap indicated row-normalized fragments per kilobase per million (FPKM) of 37 *R2R3-MYBs* from the RNA-seq database under 25°C (CK), 4°C (CT1), –20°C (CT2) in *Rosa multiflora*.

Supplementary Figure 2 The phylogenetic tree of *R2R3-MYB* genes among *Rosa multiflora*, *Rosa chinensis*, and *Arabidopsis thaliana*. Red triangle marked *R2R3-MYBs* in *R. multiflora*, green star marked *R2R3-MYBs* in *R. chinensis*, and blue circle represented *R2R3-MYB*s in *A. thaliana*.

Supplementary Figure 3 The putative regulation network of *RmMYB108* after encountering chilling in overexpressed *RmMYB108 Arabidopsis*.

Supplementary Table 1 The primers names and sequences (5′–3′) for quantitative real-time polymerase chain reaction (qRT-PCR), reverse-transcriptase polymerase chain reaction (RT-PCR), cloning, overexpression, subcellular localization, and self-activation.

Supplementary Table 2 The name, raw fragments and fragments per kilobase per million (FPKM) of *R2R3-MYBs* that respond to low temperature from transcriptome sequencing of *Rosa multiflora*.

Supplementary Table 3 The subgroups and sequences of 185 *RmMYBs* from the whole genome of *Rosa multiflora*.

Supplementary Table 4 The names, nucleotide sequences, and encoded amino acid sequences of 119 *R2R3-MYB*s in *Rosa multiflora*.

Supplementary Table 5 The nucleotide and encoded amino acid sequences of *R2R3-MYBs* in *Arabidopsis thaliana* from The Arabidopsis Information Resource (TAIR).

Supplementary Table 6 The prediction of sequence and *cis*-acting elements from PlantCARE of *AtICE1* promoter.
