# Supplementary figures and images for: An R2R3-MYB Transcription Factor RmMYB108 Responds to Chilling Stress of Rosa multiflora and Conferred Cold Tolerance of Arabidopsis
Source: Front Plant Sci. 2021 Jul 27;12:696919. doi: 10.3389/fpls.2021.696919 (PMC8353178; doi:10.3389/fpls.2021.696919)

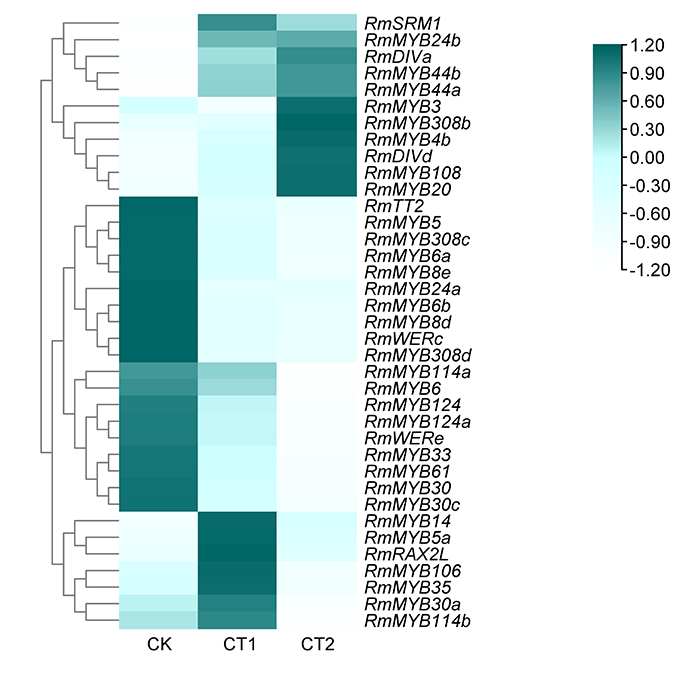

Supplement: Supplementary Figure 1 — The heatmap indicated row-normalized fragments per kilobase per million (FPKM) of 37 R2R3-MYBs from the RNA-seq database under 25°C (CK), 4°C (CT1), –20°C (CT2) in Rosa multiflora. [file Image_1.TIF]

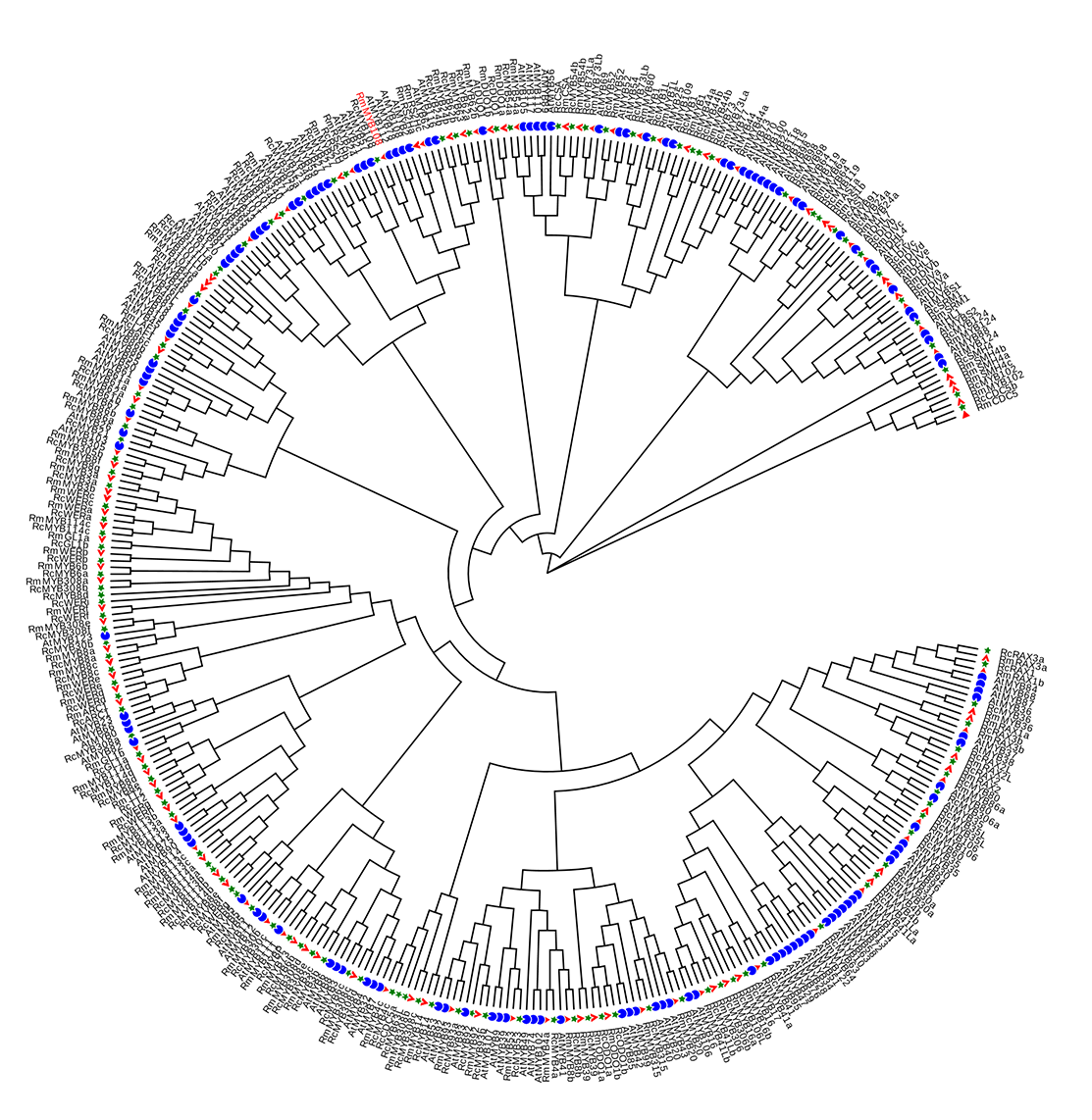

Supplement: Supplementary Figure 2 — The phylogenetic tree of R2R3-MYB genes among Rosa multiflora, Rosa chinensis, and Arabidopsis thaliana. Red triangle marked R2R3-MYBs in R. multiflora, green star marked R2R3-MYBs in R. chinensis, and blue circle represented R2R3-MYBs in A. thaliana. [file Image_2.TIF]

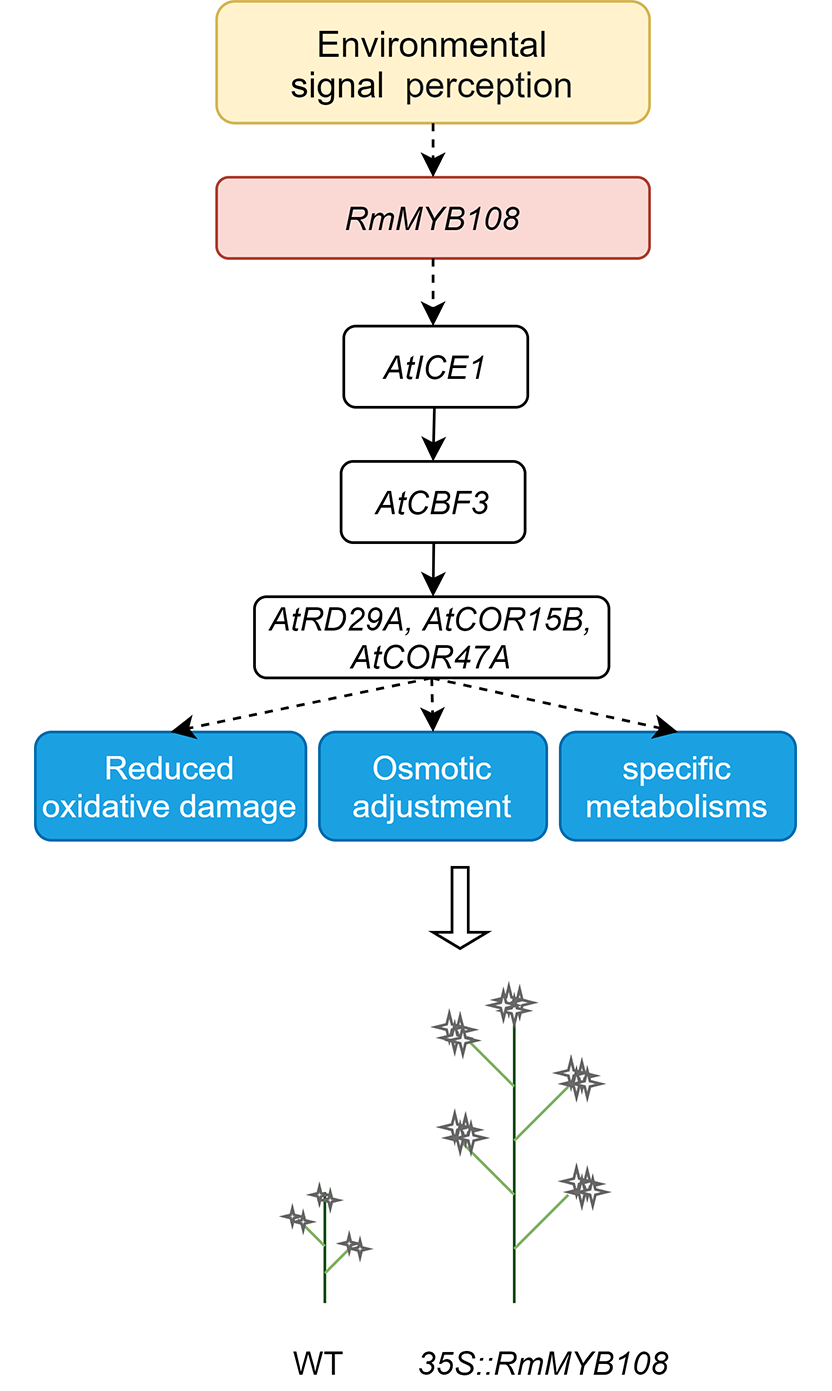

Supplement: Supplementary Figure 3 — The putative regulation network of RmMYB108 after encountering chilling in overexpressed RmMYB108 Arabidopsis. [file Image_3.TIF]
